# Concerning the Newer Development of the Pathology of Nerves, and Its Importance in Medical Instruction

**Published:** 1881-03

**Authors:** William Erb


### CONCERNING THE NEWER DEVELOPMENT OF THE PATHOLOGY OF NERVES, AND ITS IMPORTANCE IN MEDICAL INSTRUCTION.

*Delivered at the Entrance upon the Office of Professorship at the University of Leipzig, June* 16, 1880.

BY DR. WILLIAM ERB.

*Translated by Madam A. Fulgraff, D.D S., of Hamburg, Germany.*

The scientific territory of medicine has become, in our days, an extraordinarily wide field, and is rapidly progressing in extension. That which was possible to distinguished minds in former centuries, namely, to survey and even to master the whole domain of medicine, is to-day unattainable, even by the most liberal endowment and the most persevering diligence.

Since numerous jealous investigators work uninterruptedly upon natural philosophical methods, the abundance of facts in this territory has become so infinitely large that a review over the whole by *one* individual is simply impossible. The kind of subject to be worked, which often changes; the diversity of scientific objects to be managed; the manifoldness of methods and preparatory knowledge requisite for the prosperous, scientific progress, demand the confinement of the individual to certain domains of work which suit best his endowments and inclination. Therefore an extensive division of work has become absolutely necessary, and though at first it suffered very slow progress and met with resistance, it has been advancing in a more and more rapid manner, and threatens already to reach a height which gives anxiety for the future. If we look back a few decennaries and compare the few men who sufficed to make up a faculty to teach the future physician all medicine, with all her auxillary sciences, with the much larger number of those which are now thought to be indispensable for this object, and which number is probably growing, the question comes natural that if here a certain restraint should not be imposed in regard to the physician, of whom we demand a large knowledge of possibly all branches of medicine?

But as much as can be said from this point of view against the ever proceeding specialty in medicine, against the ever repeated branching of some of its single domains of knowledge and work, as little can be said against this process from the point of scientific investigation and scientific progress. The subject of work has become so enormous that a possibility of progressing consists *only* in a division of work, and in concentrating on a limited territory the ability of the individual to work. The branching of single special disciplines has to be performed with changing speed and energy, partly under the influence of some distinguished person, partly under particularly favorable circumstances.

The Pathology of the Nerves is indisputably one of those branches of clinical medicine, the development of which having made rapid progress has taken a particularly high flight. My special occupation with it for many years, justifies choosing this branch for my subject of address to-day, besides it is to be an essential part of my teaching here.

Do not expect to hear a detailed history of the modern Pathology of the Nerves. This theme would far overstep the narrow limit of my address for the present. My intention is to give you a hasty sketch of the recent development of this branch of clinical sciences. I would like to indicate briefly to you the conditions and connections which favor and make possible this very extraordinary development of the Pathology of the Nerves, and then allow me to make a few remarks upon the most expedient manner of giving instruction in this important branch, and how it ought to be conducted, according to my idea.

The last twenty years is the time in which the Pathology of the Nerves took, and still takes, a high flight in the widest sense. The number of investigators who turned to this domain grew larger from year to year; the diseases of the system of nerves became more and more interesting, when it was recognized of what great importance these diseases are not only to the physician but for solving the most difficult problems in physiology, specially in psychology and also in general pathology, not a few clinical men turned with preference to the diseases of the nerves in their clinical and experimental pathological studies, and it can almost be said that hardly any other branch of special pathology in the inner clinics is pursued with more zeal than the Pathology of the Nerves. On the other hand, a rich and extensive specialistic experience became the impulse of some physicians to make the aim of their life the investigation and treatment of the diseases of the nerves; and the furtherance of the pathology of the nerves is not a little indebted to the works of these men.

So by means of several favorable circumstances the Pathology of the Nerves has become an important branch of medical science. May I be permitted to sketch those circumstances in detail, and specially to point out the manifold, reciprocal and intimate relations which united the Pathology of the Nerves with the other branches of medical science.

The pathology of the nerves has obtained from physiology, undoubtedly the most important, and according to time, the first influence. At the beginning of the last 40 years comes that grand progress of physiology, which gained it the first rank among the biological sciences. The system of nerves was the first and main object of that vast series of exact investigations, which are the corner stones of that grand structure of the modern physiology. Supported by the progress in the science of electricity. The important investigations were executed on the motor-nerves and muscles, the knowledge of which lead to the electro-physiological laws, which have been fertile for investigating the pathology of the nerves. An entire change was made in understanding the physiology of the spinal cord; it was learned to acknowledge to this organ, which was formerly thought to be a simple collection of peripheral fibres of nerves, to be of *high importance* in the organism, and to have a comparatively high grade of independence.

Then the physiology of the organs of sense came to a high state of perfection. One began to study, by physiological methods, the connections of it to the higher psychological functions. By and by we learn to come nearer understanding the functions of the cerebrum, and to make them the object of experimental investigations. You all know how much the physiology was and is promoted by zealous working and discussing of problems; which had rested since the time of *Flourens.*

It is clear that all this was of the greatest moment to the pathology of the nerves. Questions taken up by physiology raised the interest for some other derangements of the nervous functions, which we meet in some diseases, and the interest which many distinguished physiologists took and had to take in the pathology of nerves, acted of course incitingly on pathology and produced more exact investigation and observation of diseases, having a more certain object. In many cases the obtained facts of physiology became the basis of important discoveries in the pathology of the nerves.

But pathology was not ungrateful to physiology. Physiology is indebted to pathological observation for not a few disclosures; and as there is no doubt that a large series of the most important physiological problems of the peripheral system of nerves, also of the spinal cord and especially of the brain, can only be traced to a disclosure by pathological observation on man and never by experiment on animals, so it is to be regretted that many physiologists are not only without interest for neuro-pathological facts, but have even suspicion, which is certainly not always justified.

I may refer to the fact that the neuro-pathologist was not satisfied to place his material at the disposal of the physiologists; but that not a few important physiological discoveries were made exclusively by the neuro-pathologists; partly incited by them they were made the subject of discussion. I remind, for example, the important fact of the electrical irritability of the so called motor portion of the *dura mater* which was followed by the great discussion about the localization of the function in the dura mater. I call to mind further the discovery of the reflex-phenomena coming out from the tendons and fascia; further the establishment of the laws of the galvanic irritability of the acousticus nerve; also the facts taken from pathology which speak for the functions of certain trophic nerves, etc. Also those in physiological circles so intensively discussed phenomena of the mitallascopy, of the transfer of the hypnosis, which have lately raised so much sensation, which facts have first been established exclusively by the neuro-pathologists, and scientifically treated by them.

There exists therefore between physiology and pathology a reciprocal action, which could not be more gratifying and more fruitful.

Next to physiology the perfection of the experimental pathology has been of the greatest benefit to the pathology of the nerves.

It did not remain a secret to any intelligent pathologist that here, as in all other domains of pathology, a distinguished figure is due to the experiment at least for solving certain problems, and which gives sufficent witness to the long series of experimental works presented in the neuro-pathological literature, since the celebrated investigations of *Kussmand* and *Turner* "Concerning the nature of epileptic convulsions." A great part of the physiological experiments—all those experiments of cutting and crushing the spinal cord, the extirpation and wasting of the brains and many more of such nature—is nothing but experimental pathology, and it requires a considerable amount of neuro pathological experience, in so expe.imenting, to get the right answer from nature, and to be secured from fallacy. Much, however, of which is exceedingly interesting to the pathologist of the nerves, is unfortunately not accessible, or perhaps not *yet* accessible to pathological experiment, and we are obliged to take the much (longer), more laborious and more precarious road of clinical observation, followed by pathologic anatomical investigation.

Again in the course of the fifth and sixth decades an important way was broken for progress in the pathology of the nerves frcm an other side, namely from part of a therapeutical speciality, from electro-therapeutics. Incited by the discoveries and progresses, which the use of electricity had made in the first decennaries of our century the interest of the physicans was turned more to this powerful physical agent, all the more so as a large scientific basis for these pursuits seemed to be given by the important discoveries in electro-physiology. The results surpassed the expectations, and the rapid and extensive development of the electro-therapentics of our days is known. In France it was Duchenne who introduced into the therapeutics again the faradic current; in Germany Remak, at the same time anatomist and physiologist—employ the galvanic current in a most succesful way. The therapeutic pursuits had to base on detailed physiological and neuro-pathological studies and in this way we are indebted to the works of the electro–therapeutists for an abundance of the most interesting and the most important facts for a very rich furtherance of the pathology of the nerves. The merits of Duchenne lie materially in the neuro-pathological territory: he was an observer of rare cleverness and sagacity; a clinical man in the loftest sense of the word and the important forms of diseases which he was the first to discover and to describe, perpetuate his name in the annals of science. Numerous latter investigators followed in the paths first shown by Duchenne and Remak; mainly neuro-pathological material went naturally to the electro–therapeutists, therefore they were before all others designated to observe and investigate this territory. The literature of these last two decades has taught that this has been done in an extensive manner and not without success. It is not going too far if I say that the majority of the pathologists of the nerves, at least the German pathologists, have issued from the electro-therapeutics. This is certainly a great advantage, as a complete control of the pathology of the nerves is impossible without a masterly understanding of electro-diagnostic and electro-therapeutical methods.

Lately a similar and promising relation with another branch of special therapeutics begins to develope, namely, with the Balneotherapeutics, and especially with the hydro-therapeutics. The increasing importance of the therapeutic treatment, with cold water and baths, of diseases of the nerves causes a meeting of such patients in the watering places, and will be in future a material furtherance in the pathology of the nerves. At all events the influence of this manner of cure, and the curing of nervous affections is already a very considerable one.

The beneficial influence of pathological anatomy on the pathology of the nerves was felt somewhat later than that of physiology and pathology. For the coarser conditions, however, the pathologic anatomical researches gave abundant material, and it has become here, as everywhere in pathology, a secure foundation of scientific investigation. But the pathological anatomist had only too often to be satisfied with a negative or at least absolutely insufficient statement there, where the clinical man was able to put the seat of severe deadly diseases of the nervous system with all certainty. This has been changed materially since the introduction of the modern improved investigating methods for the central system of nerves. The experienced eye sees now, according to special methods, in specimens prepared with particular care a quantity of things, the existence and signification of which we had formerly no idea, and we have now learned to get positive statements in many cases, where we received but negative results by the former methods of examining, which were also totally insufficient. The French school of the Salpetriere has been of particular importance, and to it we owe a series of beautiful discoveries; but also in Germany and England this domain was cultivated independently, from which issued many important works.

An extraordinary abundance of new and important facts were discovered in this way for the pathology of the spinal cord, which soon will be considerably augmented by the great zeal for this object existing everywhere. It may be indicated here that in this territory it has been the pathologists who, by their works, improved most the pathological anatomy of the central system of nerves, and not the professional pathologic-anatomists. To the pathologist of the nerves the most detailed knowledge of these things compose the firmest basis for further conclusions and diagnostic progresses. Repeatedly the anatomical seat of a disease was not discovered till the pathologist had said: Here it must be looked for, and nowhere else.

The possibility of such fertilizing influence of the pathological anatomy is partly explained by the great progress made in our day by the normal anatomy and the history of the development of the system of nerves. When, by the laborious and energetic investigation of Stilling, the right road was shown, by which, with purely anatomical methods, an insight is gained into the most complex construction of the central organs of the nerves. When by this method the most important outlines of the finer construction of the spinal cord, and of the brain were established, partly by Stilling, himself, and partly by his distinguished followers, yet, only then the attempt could be made with these very methods from the pathologic anatomical side to perceive and localize accurately the finer pathological lesions. Of course, also, from the anatomical part, there remains much to be done by these methods, for the infinitely complicated construction of the cerebrum would never be fully revealed if it were not possible to find new and farther reaching methods for investigation. And, indeed, such have been found already. The method of the history of the development, which at fixed times of the development, tries to gain a judgment in the construction of the central system of nerves on the ground of the successive perfection of the nervous elementary parts of different physiological diversity, on which being dependent the anatomical diversity of certain systems of fibres, a method which has been realized with great success by one of our colleagues here in

Leipzic. It has already contributed considerably to the clearing up of pathological conditions. It has also found important points of support in pathological facts, and promises for the future many more important explanations, so that we may look for its further perfection and application with great expectation.

Besides these sciences, there has been a clinical discipline of distinguished influence on the pathology of the nerves, namely, ophthalmology. It is known how extraordinarily rapid this youngest branch of medicine developed itself in the hands of ingenious masters, and under the enthusiastic work of numerous talented pupils to a hight of perfection, of which the other clinical disciples might almost be jealous. This department is indebted, before all, to this strict physiological course and method of investigation, and to the excellent improvement of the physical method of examination, and it was thus able to flourish so rapidly; and it has thus become a shining example to the other branches. By the ingenious invention of Helmholtz, the eye, this part of the central organ, and its apparatus of motion, its parts which are sensible of light, and its sensitive conducting parts, have become accessible to our direct investigation. It has also been shown, more and more clearly, what intimate relation exists between the diseases of the eye and the different kinds of diseases of the central organ, and partly of the peripheral system of nerves. By this, we pathologists of the nerves have come already so far that we cannot call a neuro-pathologic examination of an individual complete if we have not also examined exactly the functions of the eye and made an ophthalmological investigation. On the other hand, whenever it is the question to decide by a special investigation whether a disease of the eye is connected with an affection of the central nerves, the oculist often requires the support of the pathologist of the nerves. And just now a question excites not less the ophthalmological world than the neuro-pathologists, namely—in which bearing we have to put the diseases of the eye to the localization of brain affections. Also, from this side, we have approached the great question of the day, *the localization of the functions of the brain*, and it is clear that every neuro-pathologist has all reason to engage himself in detail with ophthalmology, and to follow its progress in this domain.

These are the principal and reciprocal relations which exist between the Pathology of the Nerves and the other branches of medicine, and to which is due, as far as I can see, an essential influence on the perfection of the Pathology of the Nerves. But they alone would not indeed explain the great significance which the pathology of the nerves possesses. An essential factor of it is the extraordinary, and, as it seems to me, *undoubtedly increasing frequency of the diseases of the nerves*, including the psychosis, and the extreme importance of the welfare and calamity of the individual of the family and of his entire generation. Quite an astonishing copiousness of forms of diseases of the nerves have not only been discovered by more exact clinical observation, but the absolute frequency of diseases of the nerves has increased in our days to a fairly alarming dimension; we are also able to show some of the causes to which this sad fact owes its origin.

First, there are the social circumstances which are to be charged with the enormous accumulation of great masses of people in the centres of politics, of commerce, of industry, in the large cities, with their unavoidable consequence of misery, needs, afflictions, cares of life and bad hygienic conditions: the temptation, here so easily provoked, to drunkenness, to dissolutions, to debauchery of all kinds, and the growing danger of contagion (infection) of that most dreadful of all chronic epidemics, against which the modern state still neglects with incomprehensible blindness to take the most pressing protection.

Further, it is the *conflict of life* strained to excess in our days: the necessity to over exert all strength to gain the subsistence for the individual, for the family; the growing ambition and strife, the passion for appearances and riches, which are gaining more and more ground, and the material preliminary condition for all these exertions is the too early and strained occupations of the mind, too early and continued attendance at school, the downright (dangerous) overloading with work of the pupil without regard to bodily care; also the many perversities of education, the mistakes in the private hygiene, all this contributes to augment the frequency and heaviness of the diseases of the nerves.

Not less the multiplication of the enjoyments of life, the increased passion to enjoy, the haste and excitement of modern life compared to the life of former epochs ; the general interest taken in politics, in the religious conflicts, and in many things much more; therefore we meet so many young men who have severe attacks of the nerve affections in the middle of their preparations for examination ; so many public and learned men who are ruined by the crushing burden of work; so many merchants who become the victims of nervousness and of psychosis, who, by the growing competition, are urged to a feverishly excited activity.

Far more important for the propagation of the diseases of the nerves than all this is the *heredity*, that marvelous and inexorable law of inheritability of qualities from one generation to another, to which humanity is indebted for the most beautiful blooms of mental development and perfection, but also unspeakably much need and misery, that law that can become here a blessing, there a curse. The effects of this law are in no part of the human body more distinct than in the system of the nerves ; it is an experience which can be made daily that diseases or a weakness of the nerves, either congenital or obtained, can be transferred to the descendants, often in increased proportion and in various forms ; also when these dispositions are met with on both sides of the persons who are going to be married, or when they marry exclusively in the same family, these dispositions can become more and more increased, and finally conducted to neuropathic degeneration and dying out of the families. Naturally this is not the place to explain this any more in detail.

But from all these conditions it is easily explained why, in our days, the diseases of the nerves increase, why they are accumulating especially in the large factory cities, partly in the higher classes, which are working intensively with their minds and struggling for their existence ; why, in some families the number of diseases of the nerves become larger and more severe from generation to generation ; why neuralgia exists with extraordinary frequency in some countries where a restless and exhausting business has become the rule, as for instance, in the United States. How is it that in certain races the influence of the hereditament is, so to say, involved where a great mental endowment predominates simultaneously with an extensive striving for acknowledgment and possession, and when they marry but one of their own race, and even of their own family, is why they have an extraordinary larger per centage of diseases of the nerves than others. These are facts to which we cannot be blind, and the expert eye of the pathologist of the nerves sees how the sword of Damoclese of neuro pathic degeneration threatens entire families, and whole classes of people and races. To our satisfaction, however, he sees also that nature often, in its boundless richness of power of regeneration, finds means and ways which are hidden from us, to compensate these disturbances, and how she makes good to some for the mental degeneration of their family by bestowing on them generous endowments.

All that I have mentioned has contributed to make the pathology of the nerves such as it is to-day. It would be going too far were I to sketch, even in the most superficial way, how the great progress in our knowledge has been mostly developed in detail, upon which we look with pride, without forgetting how much more and greater things are yet to be done. I cannot show in detail how in the beginning of our epoch they began to study with success particularly the diseases of neurotics, paralysis, and atrophy, and benefitted their knowledge by the various discoveries —how they learned that the causes of many diseases are central, which were hitherto taken for peripheral diseases, at the same time they confronted the central diseases with greater interest and success; in particular they began to see more clearly the diseases of the spinal cord, to arrange them in more precise forms of disease in order to trace them back to definite localization; how they came nearer to the diseases of the brain and their infinitely numerous relations to all possible functions of the body, and what progress they made lately in localizing these diseases. Even those great and marvelous neurotics have become accessible quite recently by natural, philosophical and clinical methods of searching, which from the oldest times had the bad repute of fraud, of witchcraft, of diabolism, and which seemed to mock all natural philosophical investigation. I can but hastily mention that it is a great acquisition to the modern pathology of the nerves to have found a large number of new forms of diseases; to know that the proteus-like multiformed forms of diseases as weakness of the nerves, exhaustion, hysterics and nervousness are real and very tedious diseases, the unhappy possessor of which is deeply to be pitied, whilst formerly they were generally thought to be patients with imaginary complaints, and they were wronged by incredulity and suspicion.

I must deny myself the pleasure of giving you a complete and ample picture of modern pathology of the nerves, but you will have observed from my hasty suggestions how enormously large has become the territory of the pathology of the nerves, and even if we do not count psychically, which actually belongs to it, it is anyhow one of the very voluminous chapters of special pathology. One demands, and it ought to be demanded at the present time of the pathologist of the nerves, that he know thoroughly and be able to master, besides the pathology of the nerves, the anatomy, physiology, and pathological anatomy of the system of nerves—and these are not exactly the shortest and easiest sections of this discipline—that he be perfectly at home in the neurological diagnostic and electro–therapeutics, and he must show a very large experience, which can but be obtained by laborious investigation of diseases which take up very much time, and as in the clinics or in the clinical ambulances many and practically very important forms of diseases seldom or never occur; so, we conclude the material to master completely this whole extensive territory must be taken from a large private practice.

The daily increasing stream of neuro-pathological literature—German and foreign—is almost too much for the capability of the individual, and the ideal which Griesinger has placed before our eyes, namely, that the pathologist of the nerves has to master completely the entire domain of the neurotics, including the psychosis, and with his impulse for investigation to penetrate, to work, and to teach it, is according to my idea to-day simply impossible to reach, because this task is too capacious and too divergent in different directions. A separation of these two branches, psycho-pathology and proper neuro-pathology, is imperative, and exists already almost everywhere. Of course every neuro-pathologist has to be to a certain extent psychiatist, and every psychiatist an accomplished neuro-pathologist, though the too clinical twin-sisters will meet extraordinarily often in certain boundaries of territory. They have to complete, to fertilize, and to permeate each other, but a certain independence of each cannot absolutely be refused on account of the interior reasons of the scientific topic of work, as well as the numerous exterior conditions.

It needs no further detailed explanation that the proper and possibly productive manipulation of the pathology of the nerves be to-day but a specialistic one. It requires the entire devotion, the whole working power of a man to master but in some degree this enormous territory. How much more to manipulate it independently and productively. And so that which has been performed with so many other disciplines of medicine will also be performed with the pathology of the nerves. It will branch off from the great tree of clinical sciences as a new specialistic territory of investigation and work. This branching off will be irrestible, and will take place perhaps with more right than was the case with other specialistic disciplines. For the domain of the pathology of the nerves has become so wide, the heretofore requisite special preliminary conditions, scientific and technical, so extensive that there does not remain much room for anything else.

This manipulation has to be done, according to my conviction, prevailing from a purely clinical point of view for some time yet, because we are not yet ripe for the stricter and more ideal pathologic-anatomical point of view. That one ideal pathologic-anatomical point of view, which is not at all overcome everywhere, and which, as it seems to me, is not now the most productive one for our discipline, namely, the one to which only those patients are of interest who give prospect of a speedy pathologic-anatomical investigation, and to which those forms of diseases have hardly a scientific title of existence that are not accessible to the knife and the microscope of the anatomist. I

hope nobody will believe that I underrate the high value of pathological anatomy. No one can be more convinced of its value than I, and I believe my works prove this—that the young pathologist of the nerves can do nothing more sensible than to occupy himself, if possible independently, with the pathological anatomy of the nerves, and that the pathological anatomy, as far as its capability reaches until now, will be the most excellent light in the dark of the pathology of the nerves.

But it must not, and dare not be more to the clinical man. It is in the first place but a very valuable means, and perfectly equivalent with the clinical methods of investigation for improving the scientific pathology, and for the knowledge of the laws of morbid occurrences. But the scientific pathology, has to be to the clinical man, to the genuine physician, not an exclusive aim, but a means for a higher aim. And this can be but the mutual aim of clinical disciplines, to gain from pathology, build on the ground of the exactest clinical and anatomical examination the intelligence and the means to alleviate the misery of humanity, that arises from thousands of forms of diseases.

The question where the place of this manipulation must be, is answered, of course, that it is in the clinical institutions of the universities, and in the great hospitals and infirmaries of the large cities. This mode is in Germany not, so far, or so familiar, as in other countries. The neuro-pathological or school of the Salpetriere, in Paris, has reached a high reputation under the influence of an excellent man. In England and America there exists large hospitals exclusively for patients with diseases of the nerves, which are a rich mine for experience in neuro-pathological cases. A great deal of much valuable material goes for nothing in our country, and, as is known, the psychiatry has first got so far to establish institutions exclusively for this object, and for quite different reasons than scientific ones.

The university professor has the question at heart *where and how the pathology of the nerves has to be taught*, in which way it will get a suitable place, according to its importance and difficulty, in the medical instruction. You will allow me a short answer to this question.

To me there is no doubt that the share falls to the *medical clinics* to make the young physician acquainted with the pathology and therapeutics of the system of nerves. Nobody will expect from the clinical instructor, if he himself does not feel inclined and called upon to do so, to undertake a specialistic and extensive manipulation of the pathology of the nerves, but it will be a part of his task, to give to the student of clinics, that amount of neuro-pathological knowledge which is requisite for the ordinary measure of practice, and he has to make the student acquainted with the most important and most frequently occurring forms of diseases, especially with those cases which every physician *has* to treat, which he cannot give to the specialists. Thus, the acute cases of diseases of the brain and spinal cord, graver forms of paralysis, the heavy, general neurotico, epilepsy, hysterics, etc., he has to demonstrate to the young physician *how* to make an examination in diseases of the nerves, and how to make the diagnosis, according to which principles the treatment must be given. Of course, besides the numerous and important other tasks of the inner clinics, there will remain relatively little time for all this.

I believe that much can be served here by fulfilling an essential preliminary condition: the student must—just as well here as for the other branches of internal medicine—enter the clinics with certain rudiments, before all the knowledge of the neuro-pathological methods of investigation. Then only will the instruction in clinical teaching be really fruitful; and there will be less loss of time.

The young clinician has to know before all else, the *special pathology and therapeutics of the system of nerves*; which he must have heard or studied privately. He must come to the clinics, if it would be useful to him, with a survey and systematic knowledge of it. It is unfortunately to all appearance in all universities, becoming more and more distinct, that the medical students neglect the so-called theoretical lectures; and they think they can equalize this neglect, by hearing the practical lectures. This is a thorough mistake, against which we cannot protest loudly and energetically enough, and against which some voices have already been raised. I, too, think it is truly a great damage to the modern medical perfection that this bad habit gains more and more ground, that the systematic scientific perfection is neglected in comparison to the purely practical—technical instruction, which threatens to stoop to a mere training. This creates an incompleteness and uncertainty of knowledge, against which one cannot be warned enough. The clinics and practical courses can and dare never compensate and accomplish what the modern student requires of them, indeed they become fairly injured by the student entering them with insufficient preliminary knowledge. I think an occupation with the special pathology of the nerves anticipating an entrance into the clinics is a very essential preliminary condition.

I must declare another not less essential condition which is that the student becomes closely familiar with the neuro-pathological methods of examining; and it seems to me here is a distinct gap which we must complete, in our plan of instruction. We have already had for a long time diagnostic courses for percussion and auscultation, thus for diseases of the heart and lungs; and there are at different places courses for microscopical and chemical diagnosis; but the diagnosis of the diseases of the nerves, which is at least quite as difficult, has nowhere been specially taught, as much as I know, for the electro-therepeutical courses teach but a small part of it. Such a course of the neuro-pathological diagnosis, the necessity of which has been emphsised already by *von Ziemssen*, has the aim to practice the necessary diagnostical methods of examining, (quite analagous to the courses for using percussion and auscultation, and to the courses for using the mirror for the eye and the larynx, etc.,) it has to teach the student how to test the sensibility and motility, how to examine the organs of sense and the reflections, how to manage a good electrical examination, how to test the psychial functions etc., it has to exercise the student—the entire systematic process of the neuro-pathological examination, and by practicing on patients qualify, prepare, and direct him to an independent examination as he has to execute in the clinics and poli-clinics. Such a course will, as I believe, facilitate essentially the task of the clinical man, and it can be given independently or as a part of the clinical propædeutics; this course may suffice, together with the inner clinics, to give to the young physician that degree of neuro-pathological perfection, which is *indespensable* to him.

Of course the material of the stationary clinics will by no means suffice to make the student familiar with the infinite manifoldness of forms of diseases of the nerves, which he will meet afterwards in his practice. Very numerous cases never occur in the hospitals or only in the very late stages, so all the slight cases, the many functional disturbances, the peripheral paralysis and neuralgias, the manifold general and vaso-motor neurotics, all the forms of neurastemy, the so-called nervousness, which are of enormously practical importance, the initial stages of many heavy chronic diseases of the central system of nerves, which often last for years, the recognition of which in due time is fairly of capital importance—those things are not met with in the hospitals. there now comes the poli-clinical instruction in the large ambulatories in the most satisfactory way complemental for the clinics, and I think the perfection of the physician also in this direction is one of the most important *tasks of the ambulatorial poli-clinics.*

But how and where are they to reach that degree of accomplishment? They have to possess more knowledge and more special neuro-pathological knowledge and skill than are necessary to the average physician. Of those there are not a few; and by observing more closely you will be, as I was, somewhat surprised, when I mention a large number of young physicians, to whom such special knowledge is *indespensibly* necessary.

To these belong in the first instance those physicians, who *devote themselves to the academical career and want to lecture in clinical medicine*, and who have to gather special knowledge and skill as well for the diseases of the heart and lungs for the diseases of the larynx, the skin and syphilis, as for the pathology of the nerves, if they went to work with success as clinical instructors; their number may not be very large however not every one can be automath on this territory, and many a one will be glad to find somewhere a chance to learn in less time and with less labor by able direction, which he would otherwise have gained but by diligence for years; by accidental and happy exterior circumstances, for example a situation as assistants, etc.

But then comes the very imposing number of young physicians, who want to work as *physicians aud assistants in public and private insane hospitals and in the daily increasing number of asylums for nervous patients.* He who has but somewhat of an idea of the number and size of these institutions will admit that there is an extraordinarily large proportion of such persons, who ought to know thoroughly the pathology of the nerves in fact *before* the taking of their situation.

Again, the medical leaders of the hydropathic institutions, which are of pre-eminent importance for the therapeutics of the diseases of the nerves, also the physicians of such watering places that are frequented especially by nervous patients.

Further, the physicians of large cities becoming more and more numerous, and which want to *concern themselves more particularly with the treatment of the diseases of the nerves, all the young electro-therapeutists*, who remain to become much improved in regard to their special neuro-pathological preparation and perfection, and which is at best quite as necessary as the provision of the newest electrical apparatus and the practice of the electro-therapeutical technics.

If I add to this that to every scientific ophthalmologist some special intelligence of at least some branches of the pathology of the nerves is indispensible, also a somewhat closer occupation with the pathology of the system of nerves cannot harm the young physiologist who wishes to concern himself more particularly with the physiology of the nerves. You will not doubt that alone in Germany the number of those who feel the need of more exact perfection in regard to the pathology of the nerves, can be counted by the hundreds.

Need I answer the question in an enlarged way, whether the clinical instruction is justified, or can be justified, to the wishes and wants of the young men? No; certainly not. The medical clinics have quite different, much more extensive, tasks to master. They *can not* and *dare not* have regard to these wishes and wants.

I have not the slightest doubt that a special clinical instruction in the pathology of the nerves is requisite to satisfy their wishes. The exact mode may be formed according to the existing conditions of the respective universities. Either that the clinical men who occupied themselves, with partiality, with the diseases of the nerves besides the inner clinics, (where the diseases of the nerves, of course, have to be treated in the corresponding manner to the general need) hold special clinics for the diseases of the nerves, for which suffices but a few hours a week, and so to make it possible that those students who need a special perfection can get it. Perhaps in the style of Gresinger, who used to have beside the inner clinics, psychiatrical clinics, an example of which has been followed lately by the University of Freiburg.

Or the special pathologists of the nerves give instruction in neuro-pathology on the ambulant as well as in the stationary material of diseases, and suitable to the matter at the same time in electro-therapeutics. This instruction is, at the present time, with a few exceptions, in the hands of the electro-therapeutists, that is to say, where it takes place at all. It seems to me this is not quite fit on account of the still limited circle of forms of nervous diseases; it is certainly much more efficacious that the pathologist of the nerves gives the electro-therapeutical instruction than the reverse.

The first mode may be more suitable to smaller universities in small towns, where from divers inner and outer reasons it is most suitable to the first point that the entire material of diseases be united under one direction.

The second mode is particularly recommendable for the large universities with rich material of diseases, of large means and large numbers of students. For the large universities have not only the greater number of the average physicians to teach and accomplish, but they must give to many the possibility to become accomplished, more especially and more in detail in the various directions. Hitherto this need has been fulfilled but at one large German university, and it is certainly not the smallest merit of the unforgotten *Griesinger* to have moved and executed that the clinics of nerves has become a permanent institution at the university.

The large claims on the state made by the enormous enlargement of the medical apparatus of instruction and not a little by the now everywhere existing special ophthalmological clinics and psychiatrical clinics, explains why it is more reserved in creating new institutions of instruction. But as there is no doubt that in time there will be found ways and means for this object, as it seems to me, that the mighty development and practical importance of the pathology of the nerves will give it a correspondingly large claim on a special regard. This will be the more easy, as no costly new and large institutions are requisite to establish clinics for nervous affections. Any room in a well arranged hospital is sufficient, the more so as the realization of the ambulantsic material must be regarded as of great value which has to be used for instruction in an extensive manner. Such instruction may take place with success in the large isolated hospitals, as is the case in Paris and London.

